# Prevalence and related factors of hyperuricaemia in Chinese children and adolescents: a pooled analysis of 11 population-based studies

**DOI:** 10.1080/07853890.2022.2083670

**Published:** 2022-06-13

**Authors:** Jiahuan Rao, Peiyu Ye, Jie Lu, Bi Chen, Nan Li, Huiying Zhang, Hui Bo, Xinchun Chen, Huiting Liu, Chunhong Zhang, Hua Wei, Qin Wu, Yinkun Yan, Changgui Li, Jie Mi, China Child and Adolescent Hyperuricemia Study (CCAHS) Consortium

**Affiliations:** a Center for Non-communicable Disease Management, Beijing Children’s Hospital, Capital Medical University, National Center for Children’s Health, Beijing, PR China; b Department of Endocrinology and Metabolism, Shandong Provincial Clinical Research Center for Immune Diseases and Gout, Shandong Provincial Key Laboratory of Metabolic Diseases, Qingdao Key Laboratory of Gout, the Affiliated Hospital of Qingdao University, Qingdao, PR China; c Institute of Metabolic Diseases, Qingdao University, Qingdao, PR China; d Department of Clinical Laboratory, the Second Affiliated Hospital and Yuying Children’s Hospital, Wenzhou Medical University, Wenzhou, PR China; e Tianjin Women’s and Children’s Health Center, Tianjin, PR China; f Department of Children’s and Adolescent Health, Public Health College, Harbin Medical University, Harbin, PR China; g Department of Pediatrics, Jinghai District Hospital, Jinghai Clinical College, Tianjin Medical University, Tianjin, PR China; h Department of Pediatrics, Tangshan People’s Hospital, Tangshan, PR China; i Department of pediatric, The First Hospital of Jinzhong, Shanxi, PR China; j Cancer Center, The Third Affiliated Hospital of Kunming Medical University, Yunnan Cancer Hospital, Yunnan, PR China; k Division of Rheumatology, Clinical Medical College, Yangzhou University, Jiangsu, PR China

**Keywords:** Children, adolescents, uric acid, hyperuricaemia, prevalence

## Abstract

**Background and aims:**

Hyperuricaemia can lead to gout and is associated with an increased risk of cardiometabolic disease. We aimed to investigate the prevalence of hyperuricaemia and its related factors in Chinese children and adolescents.

**Methods:**

We pooled data from 11 population-based studies comprising 54,580 participants aged 3–19 years. The sex- and age-standardized prevalence of hyperuricaemia was estimated overall and by sex, age, weight status, geographic region and survey year.

**Results:**

Serum uric acid (SUA) increased gradually from 3 to 11 years with no significant sex difference, and then increased dramatically during 11–15 years. The estimated overall prevalence of hyperuricaemia was 23.3% (26.6% in boys and 19.8% in girls, *p* < .001). The prevalence increased with growing age (3.7, 9.8, 15.8, 35.5 and 31.7% among children aged 3–5, 6–8, 9–11, 12–15 and 16–19 years, respectively, *p* for trend < .001) and with increasing weight status (18.2, 37.6, 50.6 and 64.5% among children with non-overweight, overweight, obesity and extreme obesity, respectively, *p* for trend < .001). The prevalence was higher in North than in South (24.2 *vs*. 19.7%, *p* < .001), and increased markedly from 16.7% during 2009–2015 to 24.8% during 2016–2019. In multivariable regression analyses, sex, age, obesity, region and survey year were independently associated with odds of hyperuricaemia.

**Conclusions:**

The prevalence of hyperuricaemia in Chinese children and adolescents is unexpectedly high. The findings suggest an urgent need to implement effective interventions to reduce risk of hyperuricaemia in Chinese youths.

**KEY MESSAGES**
- **Question:** What is the prevalence of hyperuricaemia in Chinese children and adolescents?
- **Findings:** In this large pooled cross-sectional study comprising >50,000 children and adolescents aged 3–19 years, we found that the prevalence of hyperuricaemia was high in overall population and subgroups of sex, age, obesity, region and survey year.
- **Meaning:** Our findings indicate that hyperuricaemia is an important health problem in Chinese children and adolescents, and effective intervention strategies are needed to reduce its burden.

## 1. Introduction

Serum uric acid (SUA) is the ultimate metabolite of purine. Hyperuricaemia is caused by urine metabolism disorder that is the interaction result of many factors including high purine diet and obesity [1]. There is convincing evidence that hyperuricaemia is an established risk factor for gout and nephrolithiasis [2]. Several longitudinal studies have shown that elevated SUA levels are associated with increased risks of future renal dysfunction [3], type 2 diabetes [4] and adverse cardiovascular outcomes [5].

With the rapid socio-economic development of China in recent decades, purine-abundant diets and physical inactivity have become increasingly common [6,7], which may lead to increasing prevalence of hyperuricaemia [8]. A meta-analysis of adults showed that the prevalence of hyperuricaemia was 13.3% in mainland China during 2000–2014 [9]. However, the prevalence of hyperuricaemia among Chinese children and adolescents is poorly characterized. Previous cross-sectional studies have demonstrated that the prevalence of hyperuricaemia in Chinese youths varied across surveys ranging from 10.1 to 25.4% [10–19], and most of these studies are limited to certain area, small sample size, specific age period or inconsistent diagnostic criteria. Therefore, it is particularly important to obtain national prevalence and related factors, which can help to improve health awareness and formulate appropriate public health policies. In this study, we aimed to estimate the prevalence of hyperuricaemia and identify its related factors in Chinese children and adolescents during 2009–2019 using pooled sample from 11 population-based studies.

## 2. Methods

### 2.1. Study population

We pooled sample of 58,993 children and adolescents from 11 population-based studies conducted during 2009–2019. Detailed information of these studies has been previously published [10–20]. These studies involved 14 provinces or municipalities and the sample size ranged from 509 to 21,602. Participants were recruited from communities in six studies, schools in three studies or health examination centres in two studies (Table 1). Data on demographic characteristics, anthropometric parameters, socioeconomics and SUA were obtained from each study. We finally included a total of 54,580 participants for analysis after excluding those who: 1) had missing data for sex or age (*n* = 901); 2) were aged < 3 years (2987) or > 19 years (*n* = 525); or 3) had genetic diseases or acute or serious chronic diseases (Supplemental Figure 1). Each study had been approved by their respective Institutional Ethics Review Board.

**Table 1.** Characteristics of included studies.

Author	Survey year	Province	Region	Age, year	Sample size	Boy, %	Population source	Specimen	Laboratory method
CHNS [20]	2009	9 Provinces^a^	South + North	3–19	907	54.7	Community	Serum	Enzymatic colorimetric
Bo et al. [10]	2010	Tianjin	North	7–17	1269	50.3	Community	Serum	Enzymatic colorimetric
Chen et al. [11]	2012	Hebei	North	4–15	999	56.0	Health examination centre	Serum	Enzymatic colorimetric
Liu et al. [12]	2013	Shanxi	North	8–14	809	52.8	School	Serum	Enzymatic colorimetric
Zhuang et al. [13]	2014	Heilongjiang	North	10–18	1640	49.8	Community	Serum	Enzymatic colorimetric
Li et al. [14]	2015	Tianjin	North	3–6	4073	52.5	Community	Serum	Enzymatic colorimetric
Zhang et al. [15]	2015	Nationwide	South + North	17–19	600	100	School	Serum	Enzymatic colorimetric
Lu et al. [16]	2017	Shandong	North	13–19	21,602	49.1	Community	Serum	Enzymatic colorimetric
Chen et al. [17]	2017	Zhejiang	South	3–19	10,764	61.6	Health examination centre	Serum	Enzymatic colorimetric
Wu et al. [18]	2018	Jiangsu	South	13–16	509	49.1	Community	Serum	Enzymatic colorimetric
Ye et al. [19]	2019	Beijing	North	6–16	11,408	49.9	School	Serum	Enzymatic colorimetric

^a^Including Heilongjiang, Liaoning, Shandong, Jiangsu, Guangxi, Guizhou, Henan, Hubei and Hunan.

### 2.2. Measurements

Weight and height were measured twice with lightweight clothing and no shoes in all studies. Body mass index (BMI) was calculated as weight in kilograms divided by the square of height in metres. SUA was measured using the automatic analyser based on the enzymatic colorimetric method in all studies.

### 2.3. Definitions

Weight status for children aged 3–19 years were classified as non-overweight, overweight and obesity according to sex- and age-specific BMI cut-off values recommended by the International Obesity Task Force (IOTF) [21]. Extreme obesity was defined according to 1.2 times of BMI cut-off values for obesity [22]. For those ≥18 years, overweight and obesity were defined using cut-off points of 25 and 30 kg/m^2^, respectively [23]. Hyperuricaemia was defined as SUA > 420 μmol/L (7 mg/dL) in boys and >360 μmol/L (6 mg/dL) in girls [24].

### 2.4. Statistical analysis

The prevalence rates were sex- and age-standardized according to the Sixth National Census. We estimated the prevalence in overall population and among subgroups of sex, age period, weight status, survey year and region. The current guideline for the diagnosis and management of hyperuricaemia and gout in China recommends that urate-lowering drugs should be used immediately regardless of the symptoms if SUA ≥ 540 μmol/L [25]. Thus, we also estimated the prevalence of children with SUA ≥ 540 μmol/L. Chi-square tests were used to compare the prevalence among subgroups. Multivariable logistic regression analyses were used to identify related factors associated with risk of hyperuricaemia. We stratified China into North and South by Qinling–Huaihe Line (longitude 104°15′E − 120°21′E and latitude 32°18′N − 34°05′N) to investigate whether the prevalence of hyperuricaemia varies across regions (Supplemental Table 1).

All data analyses were performed by IBM SPSS Statistics for Windows version 23.0 (IBMCorp., Armonk, NY). A two-sided *p* value <.05 was considered statistically significant.

## 3. Results

### 3.1. Characteristics of the participants

A total of 54,580 children and adolescents (boys, 52.9%) aged 3–19 years were included (Table 2). Participants aged 12–15 years accounted for 21.9%, and those aged 16–19 years accounted for 37.9%. The prevalence of overweight and obesity was 15.3 and 6.8%, respectively. Most participants were from the North (77.4%) and surveyed during 2016–2019 (81.1%).

**Table 2.** Characteristics of study population.

	Overall (*N* = 54,580)	Boys (*N* = 28,849)	Girls (*N* = 25,731)
Age (years)			
** **3–5	5896 (10.8)	3410 (11.8)	2486 (9.7)
** **6–8	9025 (16.5)	4887 (16.9)	4138 (16.1)
** **9–11	7010 (12.8)	3842 (13.3)	3168 (12.3)
** **12–15	11,980 (21.9)	6258 (21.7)	5722 (22.2)
** **16–19	20,669 (37.9)	10,452 (36.2)	10,217 (39.7)
Weight status^a^			
** **Non-overweight	36,433 (77.9)	17,720 (73.1)	18,713 (83.0)
** **Overweight	7161 (15.3)	4276 (17.7)	2885 (12.8)
** **Obesity	2639 (5.6)	1831 (7.6)	808 (3.6)
** **Extreme obesity	539 (1.2)	399 (1.6)	140 (0.6)
Region			
South	12,326 (22.6)	7667 (26.6)	4659 (18.1)
North	42,254 (77.4)	21,182 (73.4)	21,072 (81.9)
Survey year			
** **2009–2015	10,297 (18.9)	5676 (19.7)	4621 (18.0)
** **2016–2019	44,283 (81.1)	23,173 (80.3)	21,110 (82.0)

^a^The numbers of subjects with missing values were 7808 for height or weight.

### 3.2. Sex- and age-specific level of serum uric acid

**Figure 1.**
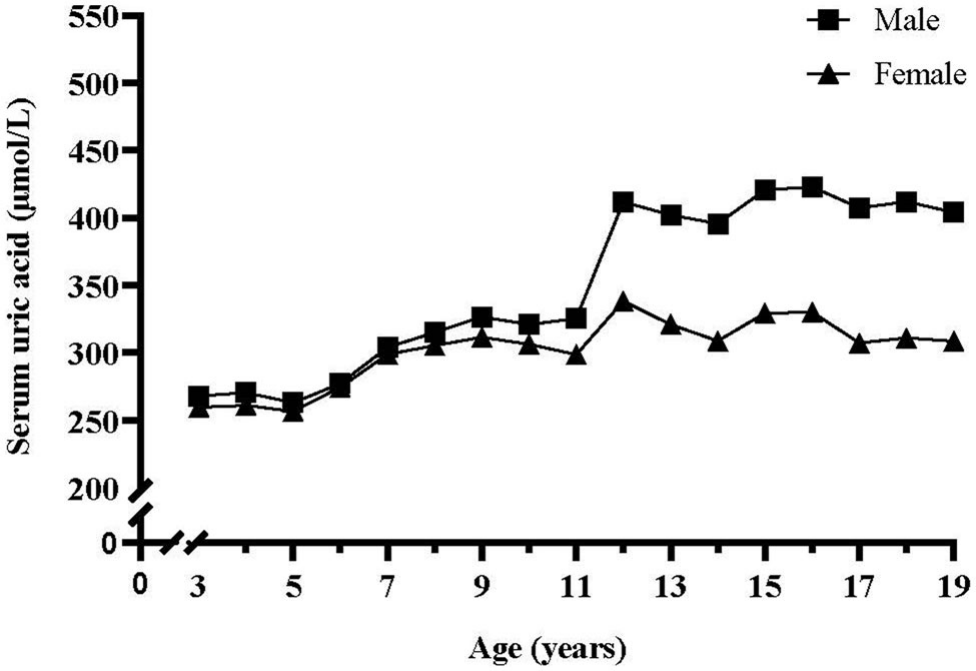
Change in mean serum uric acid with age by sex.

Figure 1 presents the change curves of SUA during 3–19 years by sex. Boys *vs.* girls showed similar change patterns. SUA increased gradually from 3 to 11 years with no significant sex difference; then SUA increased dramatically around 11–15 years when SUA reached adult levels, but boys had a more rapid increase during this period; from 15 to 19 years, SUA remained almost unchanged for both sexes, but boys had significantly higher SUA levels than girls.

### 3.3. Prevalence of hyperuricaemia

**Figure 2.**
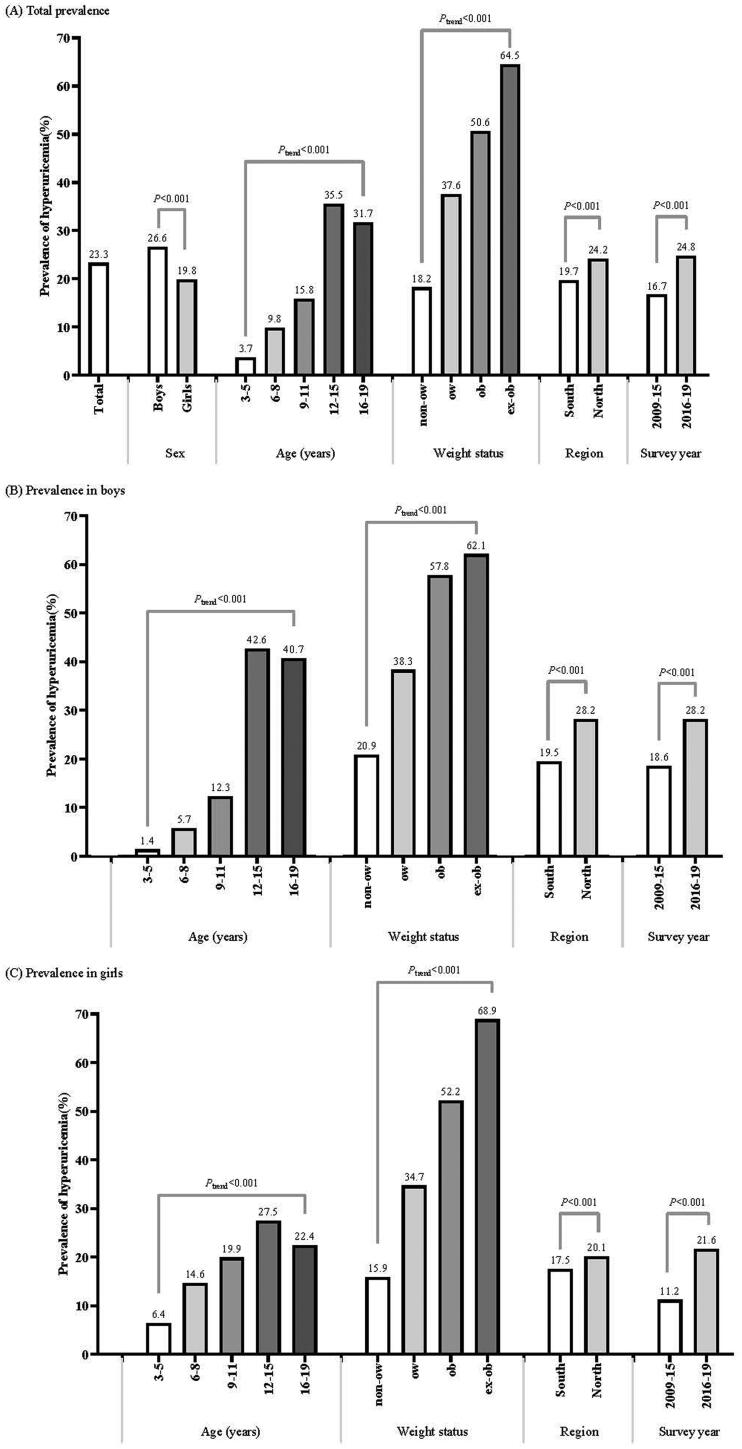
Prevalence of hyperuricaemia among Chinese children and adolescents. non-ow: non-overweight; ow: overweight; ob: obesity; ex-ob: extreme obesity.

The overall estimated prevalence of hyperuricaemia was 23.3% (26.6% in boys and 19.8% in girls, *p* < .001). The prevalence of hyperuricaemia gradually increased with age and peaked at 12–15 years old (35.5%). Compared with non-overweight children, the prevalence of hyperuricaemia in overweight children (37.6%), obese children (50.6%) and extremely obese children (64.5%) was significantly higher (*p* for trend < .001). The prevalence in the North was higher than that in the South (24.2 *vs.* 19.7%, *p* < .001) and increased significantly over the past decade from 16.7% in 2009–2015 to 24.8% in 2016–2019 (*p* < .001). Similar trends were found among subgroups of sex (Figure 2 and Supplemental Table 2) and region (Supplemental Table 3). Of note, 2.9% of children and adolescents had a SUA level of ≥ 540 μmol/L (boys, 5.0%; girls, 0.5%) (Supplemental Table 4).

### 3.4. Related factors of hyperuricaemia

In the overall population, after adjustment for covariates, boys *vs.* girls conferred an increased odds of hyperuricaemia (OR = 1.54, 95% CI = 1.47–1.61). Increasing age was associated with increased odds of hyperuricaemia, and the ORs and 95% CIs were 4.05 (3.77–4.35) for 12–15 years and 3.65 (3.41–3.91) for 16–19 years, respectively. The odds of hyperuricaemia increased with obesity severity, and the ORs and 95% CIs were 2.99 (2.82–3.16) for overweight, 5.40 (4.93–5.91) for obesity and 9.76 (8.03–11.87) for extreme obesity, respectively. Moreover, the odds of hyperuricaemia were higher in the North than South, higher in 2016–2019 than 2009–2015 (Table 3). Similar results were found in both boys and girls.

**Table 3.** Odds ratios (95% confidence intervals) for hyperuricaemia associated with related factors.

	Overall		Boys		Girls	
Characteristics	Model 1	Model 2	Model 1	Model 2	Model 1	Model 2
Sex						
Girl	Ref	Ref	–	–	–	–
Boy	1.44 (1.38–1.50)	1.54 (1.47–1.61)	–	–	–	–
Age						
3–11	Ref	Ref	Ref	Ref	Ref	Ref
12–15	5.16 (4.87–5.47)	4.05 (3.77–4.35)	11.06 (10.13–12.08)	9.19 (8.25–10.23)	2.39 (2.20–2.59)	1.67 (1.51–1.85)
16–19	4.16 (3.95–4.39)	3.65 (3.41–3.91)	9.87 (9.10–10.72)	9.10 (8.21–10.09)	1.70 (1.58–1.83)	1.33 (1.21–1.46)
Weight status						
Non-overweight	Ref	Ref	Ref	Ref	Ref	Ref
Overweight	2.85 (2.70–3.01)	2.99 (2.82–3.16)	2.68 (2.50–2.87)	2.93 (2.71–3.17)	2.88 (2.65–3.13)	2.98 (2.72–3.26)
Obesity	4.38 (4.05–4.75)	5.40 (4.93–5.91)	3.56 (3.22–3.92)	5.67 (5.03–6.39)	5.62 (4.87–6.49)	6.38 (5.48–7.42)
Extreme obesity	7.05 (5.91–8.42)	9.76 (8.03–11.87)	5.70 (4.64–7.01)	11.53 (9.03–14.74)	9.22 (6.50–13.08)	11.77 (8.13–17.03)
Region						
South	Ref	Ref	Ref	Ref	Ref	Ref
North	2.69 (2.54–2.85)	1.55 (1.39–1.72)	3.96 (3.67–4.28)	1.52 (1.32–1.74)	1.66 (1.52–1.81)	1.52 (1.28–1.80)
Survey year						
2009–2015	Ref	Ref	Ref	Ref	Ref	Ref
2016–2019	2.90 (2.72–3.09)	2.35 (2.19–2.53)	3.02 (2.78–3.28)	2.13 (1.94–2.35)	2.83 (2.55–3.14)	3.03 (2.70–3.41)

Model 1: Univariate logistic regression analysis.

Model 2: Multivariate logistic regression analysis adjusted for sex, age period, weight status, region and survey year.

## 4. Discussion

This large pooled cross-sectional study reported the overall prevalence and related factors of hyperuricaemia in Chinese children and adolescents aged 3–19 years and found high prevalence in the overall population and subgroups. Moreover, sex, age, weight status, region and survey year were independent factors related to risk of hyperuricaemia.

Previous studies have demonstrated that the prevalence of hyperuricaemia in children and adolescents varied across countries. A nationally representative subsample from 2013 to 2016 US National Health and Nutrition Examination Survey showed that the weighted prevalence of hyperuricaemia was 16.56% among adolescents aged 12–19 years [26]. Another cross-sectional survey using data from the 7th Korea National Health and Nutrition Examination Survey (2016–2017) reported that the prevalence of hyperuricaemia was 9.4% (male, 8.4%; female, 10.5%) among children aged 10–18 years [27]. In contrast, our study found that the prevalence in Chinese children and adolescents was 23.3%, which was much higher than that in other countries. This difference may be due to difference in ethnic, obesity epidemic, eating habits and lifestyle [28,29]. Of note, we found that 2.9% of children had a SUA level of higher than 540 μmol/L (Supplemental Table 4), which means they need to receive uric acid-lowering therapy according to Chinese clinical guidelines [25]. Our findings suggest that the high prevalence of hyperuricaemia in children and adolescents in China has become a public health problem that cannot be ignored.

The increasing consumption of high purine diet may be one of the reasons explaining the high prevalence of hyperuricaemia among Chinese youths observed in our study. In the past several decades, rapid urbanization in China has led to changes from traditional to Western diets characterized by a large proportion of animal source foods with high purine [30]. Fructose is a popular dietary ingredient and widely used in sugar-sweetened beverages, and there is compelling evidence that fructose-containing sweeteners and drinks are associated with increased risk of hyperuricaemia by stimulating the catabolism of adenine nucleotides [31]. The consumption of sugar-sweetened beverages has progressively increased more than tenfold from 2003 to 2014 in children and adolescents [32]. In parallel with the trend of these dietary factors, we found that the prevalence of hyperuricaemia increased from 16.7% during 2009–2015 to 24.8% during 2016–2019. Unfortunately, data on diet are unavailable in our study and the effect of diet needs to be explored further. Several randomized clinical trial studies have demonstrated that low-purine diet and DASH diet can effectively lower SUA among participants with hyperuricaemia [33], but few studies have focussed on diet intervention on child hyperuricaemia. Thus, future studies are required to explore the feasibility of dietary intervention strategies for children with hyperuricaemia.

Consistent with previous findings, our study found that obesity was associated with increased risk of hyperuricaemia in children and adolescents. Previous studies have reported that the prevalence of overweight and obesity increased from 17.1% in 2010 to 22.5% in 2014 [34]. In addition, the prevalence of obesity in children in the north is significantly higher than that in the south [35], and similar results were found in our study (23.3% in the north *vs*. 11.7% in the south) (Supplemental Table 5). The regional difference in obesity prevalence may partly explain the finding that hyperuricaemia was more prevalent in North. Obesity is thought to be the cause of hyperinsulinemia, and the physiological mechanisms mainly involve dysregulation of lipid, insulin resistance, inflammation and adipokines imbalance [36–38]. Therefore, maintaining the ideal weight or guiding obese children to lose weight is conducive to reduce the risk of hyperuricaemia.

In this study, we found that SUA experienced a gradual increase before 11 years for both sexes. SUA underwent a rapid increase around puberty with boys showing a more rapid increase, and thus sex difference begins to appear. One previous study of children aged 1–19 years in Taiwan reported similar results [39]. The underlying mechanisms for sex difference in SUA levels remain unclear, but this physiological difference may be partially explained by the action of sex hormones, such as oestrogen on the xanthine oxidase activity and the renal excretion of uric acid [40]. Muscle tissue is a major site of de novo purine production in the body, and muscle mass increase more rapidly in boys than in girls during puberty [41]. Thus, the difference in muscle mass between boys and girls may be another potential explanation. Sex- and age-specific reference intervals for SUA have been established in US children and adolescents to deal with the fluctuation of SUA during childhood [42]. Given race/ethnic difference in SUA levels, it is of great importance to establish sex- and age-specific references for Chinese children and adolescents in the future study.

The major strength of our study is that this large mixed sample comprising >50,000 children and adolescents aged 3–19 years, allowing for stratified analyses by multiple factors. All studies applied rigorous quality control procedures, including standardized anthropometric and SUA measurement. However, several limitations should be noted. First, our participants were predominantly from the North and recruited during 2016–2019, and the number of participants from south region and before 2016 were small. Second, unhealthy lifestyle including diet and physical activity is an established risk factor for hyperuricaemia; however, data on lifestyle are unavailable in our study. Third, we used adult SUA cut-points for defining hyperuricaemia, which may be not appropriate for children and adolescents. Further study is necessary to establish age- and sex- specific SUA cut-points in children and adolescents.

## 5. Conclusion

In this large pooled sample in China, the prevalence of hyperuricaemia is unexpectedly high in children and adolescents aged 3–19 years. Sex, age and obesity are important risk factors of hyperuricaemia. These findings have important public health implications for implementing effective interventions to reduce the risk of hyperuricaemia in Chinese youths.

## Supplementary Material

Supplemental Material

## Data Availability

The data that support the findings of this study are available from the corresponding author upon reasonable request.
