# Supplementary material for: Prevalence and related factors of hyperuricaemia in Chinese children and adolescents: a pooled analysis of 11 population-based studies
Source: Ann Med. 2022 Jun 13;54(1):1608–15. doi: 10.1080/07853890.2022.2083670 (PMC9225777; doi:10.1080/07853890.2022.2083670)
Supplement: Supplemental Material [file IANN_A_2083670_SM9231.docx]

**Prevalence and related factors of hyperuricemia in Chinese children and adolescents: a pooled analysis of 11 population-based studies**

**Appendices**

**Supplemental Table 1.** Geographical division of mainland China

**Supplemental Table 2.** Prevalence of hyperuricemia among Chinese children and adolescents

**Supplemental Table 3.** Stratified analysis for the prevalence of hyperuricemia among Chinese children and adolescents

**Supplemental Table 4.** Percentage of serum uric acid greater than 540μmol/L in Chinese children and adolescents

**Supplemental Table 5.** Prevalence of obesity in Chinese children and adolescents

**Supplementary Figure 1.** Study flow diagram

**Supplemental Table 1. Geographical division of mainland China^*^**

|  | Province or municipality |
| --- | --- |
| North | **Beijing**, **Tianjin**, **Liaoning**, **Shandong**, **Hebei**, **Shanxi**, Jilin, **Heilongjiang**, **Henan**, Inner Mongoria, Shaanxi, Gansu, Qinghai, Ningxia, Xinjiang |
| South | Shanghai, **Jiangsu**, **Zhejiang**, Fujian, Guangdong, Anhui, Jiangxi, **Hubei**, Hunan, Hainan, **Guangxi**, Chongqing, Sichuan, **Guizhou**, **Yunnan**, Tibet |

**^*^** The main source of participants in this study was shown in bold.

**Supplemental Table 2. Prevalence of hyperuricemia among Chinese children and adolescents^*^**

|  | **Overall** |  | **Boys** |  | **Girls** |
| --- | --- | --- | --- | --- | --- |
| **Overall, (%)** | 23.8/23.3 |  | 26.8/26.6 |  | 20.3/19.8 |
| **Age(years)** |  |  |  |  |  |
| 3-5 | 3.3/3.7 |  | 1.3/1.4 |  | 6.0/6.4 |
| 6-8 | 9.4/9.8 |  | 5.3/5.7 |  | 14.1/14.6 |
| 9-11 | 16.1/15.8 |  | 12.5/12.3 |  | 20.4/19.9 |
| 12-15 | 36.1/35.5 |  | 43.4/42.6 |  | 28.2/27.5 |
| 16-19 | 31.3/31.7 |  | 40.6/40.7 |  | 21.8/22.4 |
| *P*_trend_ Value | <0.001 |  | <0.001 |  | <0.001 |
| **Weight status^#^** |  |  |  |  |  |
| Non-overweight | 19.8/18.2 |  | 22.9/20.9 |  | 16.8/15.9 |
| Overweight | 41.3/37.6 |  | 44.3/38.3 |  | 36.7/34.7 |
| Obesity | 51.9/50.6 |  | 51.4/57.8 |  | 53.1/52.2 |
| Extreme obesity | 63.5/64.5 |  | 62.9/62.1 |  | 65.0/68.9 |
| *P*_trend_ Value | <0.001 |  | <0.001 |  | <0.001 |
| **Region** |  |  |  |  |  |
| South | 12.2/19.7 |  | 10.9/19.5 |  | 14.3/17.5 |
| North | 27.1/24.2 |  | 32.6/28.2 |  | 21.6/20.1 |
| *P* Value | <0.001 |  | <0.001 |  | <0.001 |
| **Survey year** |  |  |  |  |  |
| 2009-2015 | 11.2/16.7 |  | 12.6/18.6 |  | 9.4/11.2 |
| 2016-2019 | 26.7/24.8 |  | 30.3/28.2 |  | 22.7/21.6 |
| *P* Value | <0.001 |  | <0.001 |  | <0.001 |

^*^ Data was shown in crude rate/adjusted rate. Sex- and age-adjusted rates were calculated using the urban population composition of the Sixth National Census in China (direct standardization).

^#^ The numbers of subjects with missing values were 7808 for height or weight.

**Supplemental Table 3.** **Stratified analysis for the prevalence of hyperuricemia among Chinese children and adolescents^*^**

|  | **South** | |  | **North** | |
| --- | --- | --- | --- | --- | --- |
|  | **N** | **Prevalence** |  | **N** | **Prevalence** |
| **Weight status** |  |  |  |  |  |
| Non-overweight | 4,161 | 8.7/14.2 |  | 32,272 | 21.2/18.6 |
| Overweight | 460 | 18.0/31.3 |  | 6,701 | 42.8/38.1 |
| Obesity^#^ | 94 | 29.8/56.0 |  | 3,084 | 54.6/52.6 |
| **Survey year** |  |  |  |  |  |
| 2009-2015 | 1,053 | 21.7/20.3 |  | 9,244 | 10.0/17.0 |
| 2016-2019 | 11,273 | 11.3/22.3 |  | 33,010 | 32.0/31.1 |

^*^ Data was shown in crude rate/adjusted rate. Sex- and age-adjusted rates were calculated using the urban population composition of the Sixth National Census in China (direct standardization).

^#^ Including obesity and extreme obesity.

**Supplemental Table 4. Percentage of serum uric acid greater than 540μmol/L in Chinese children and adolescents**

|  | **≥540μmol/L (%)** | **<540μmol/L (%)** |
| --- | --- | --- |
| **Total** | 2.9 | 97.1 |
| **Boys** | 5.0 | 95.0 |
| **Girls** | 0.5 | 99.5 |

**Supplemental Table 5. Prevalence of obesity in Chinese children and adolescents**

|  | **Overweight** | |  | **Obesity^*^** | |
| --- | --- | --- | --- | --- | --- |
|  | **N** | **Prevalence** |  | **N** | **Prevalence** |
| **Region** |  |  |  |  |  |
| South | 460 | 9.8 |  | 94 | 2.0 |
| North | 6,701 | 15.9 |  | 3,084 | 7.3 |
| **Survey year** |  |  |  |  |  |
| 2009-2015 | 1,471 | 14.4 |  | 760 | 7.4 |
| 2016-2019 | 5,690 | 15.6 |  | 2,418 | 6.6 |

^*^ Including obesity and extreme obesity.

**
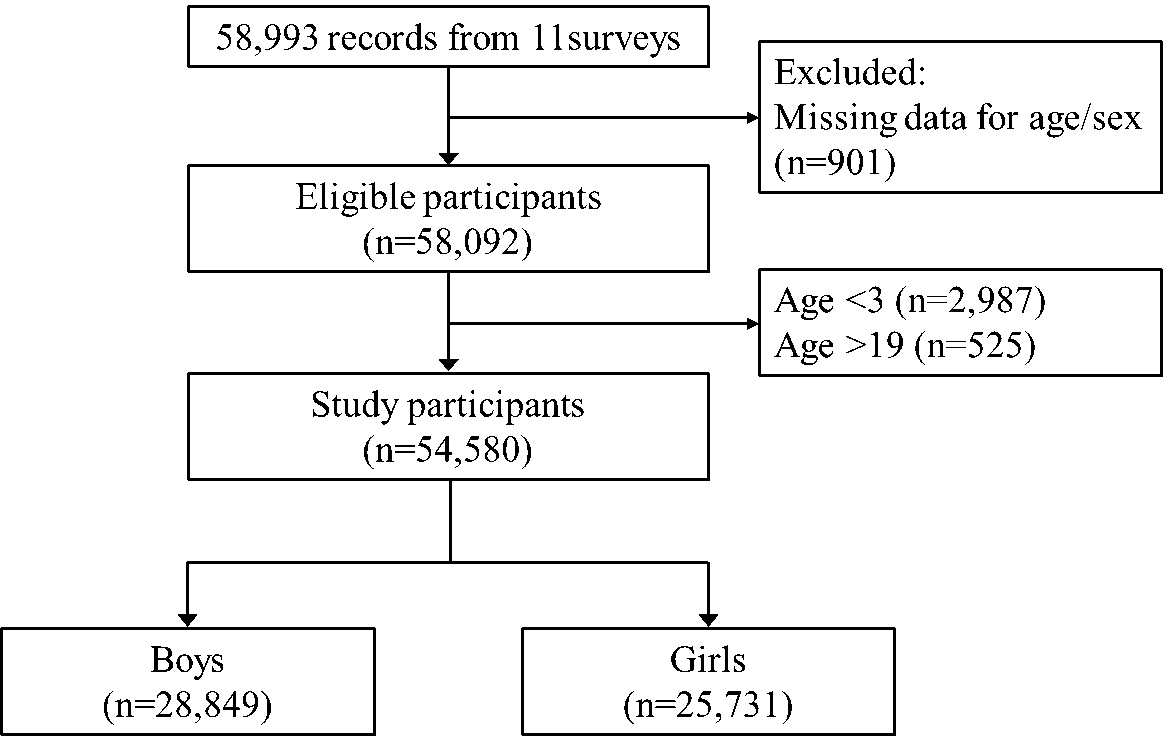
**

**Supplemental Figure 1. Study flow diagram**
